# Muscle Loss in Obesity Therapy as a Therapeutic Target: Trial Design and Endpoints for Regulatory Discussions

**DOI:** 10.1002/jcsm.70147

**Published:** 2025-12-08

**Authors:** Stephan von Haehling, Ryosuke Sato, Henning Langer, Muhammad Shahzeb Khan, Andrew J. S. Coats, William Evans, Steven Heymsfield, Stefan D. Anker

**Affiliations:** ^1^ Department of Cardiology and Pneumology University of Göttingen Medical Center Göttingen Germany; ^2^ German Center for Cardiovascular Research (DZHK), Partner Site Lower Saxony Göttingen Germany; ^3^ Department of Geriatrics and Medical Gerontology, Charité–Universitätsmedizin Berlin Corporate Member of Freie Universität Berlin and Humboldt‐Universität zu Berlin Berlin Germany; ^4^ Baylor Scott and White Health‐The Heart Hospital Plano Texas USA; ^5^ Baylor Scott and White Research Institute Dallas Texas USA; ^6^ Heart Research Institute University of Sydney Sydney Australia; ^7^ Department of Nutritional Sciences and Toxicology University of California Berkeley Berkeley California USA; ^8^ Division of Geriatrics Duke University Medical Center Durham North Carolina USA; ^9^ Pennington Biomedical Research Center Louisiana State University Baton Rouge Louisiana USA; ^10^ Department of Cardiology (CVK) German Heart Center Charité Berlin Germany; ^11^ German Centre for Cardiovascular Research (DZHK) Partner Site Berlin Charité Universitätsmedizin Berlin Germany

**Keywords:** clinical trials, diagnostics, endpoints, GLP‐1RA, muscle wasting, obesity, patient reported outcomes, sarcopenia, treatment

## Abstract

The Society on Cachexia and Wasting Disorders (SCWD) convened a Regulatory and Trial Update Workshop in Washington, D.C., in December 2024, assembling experts from academic institutions, the pharmaceutical industry and the US Food and Drug Administration (FDA) for focused discussions. This article summarizes the latter half of the meeting, which primarily focused on novel anti‐obesity therapies based on incretin pathway alteration. Discussions highlighted the impact of glucagon‐like peptide‐1 (GLP‐1) receptor agonists or GLP‐1/glucose‐dependent insulinotropic polypeptide (i.e., GLP‐1/GIP) agonists on body composition and muscle health; the challenges of distinguishing ‘true’ skeletal muscle from fat‐free tissue; the impact of treatment discontinuation and weight regain; advances in imaging and quantitative assessment of lean body mass; as well as insights from emerging muscle‐preserving therapies (e.g., bimagrumab, pemvidutide and enobosarm). There are significant challenges in defining meaningful structural, functional and patient‐reported endpoints for the use of muscle‐‘protective’ drug therapies in the context of weight loss therapies. These also involve significant regulatory considerations for future drug development and approval pathways, for instance related to the very large number of individuals that may be considered for these therapeutic approaches as well as from the potential long (or life‐long) duration of therapy considered with these drugs. Together, these discussions highlight the growing importance of integrating body composition and functional assessments in future clinical trials.

## 1 Introduction

The Society on Cachexia and Wasting Disorders (SCWD) convened a Regulatory and Trial Update Workshop in Washington, D.C., in December 2024, assembling experts from academic institutions, the pharmaceutical industry and the US Food and Drug Administration (FDA) for focused discussions. This article summarizes the latter half of the meeting, which primarily centred on novel anti‐obesity therapies based on incretin pathway alteration.

## 2 Incretins and Muscle Loss

Dr. Langer opened the session about the potential effects of incretin therapy on skeletal muscle by highlighting the lean tissue loss data from the STEP 1 trial [1]. The trial, which led to the approval of semaglutide for obesity treatment, revealed that up to 40% of total body weight loss could be attributed to lean body mass reduction, exceeding the approximate 25% commonly seen with standard caloric restriction [2]. This unexpected result prompted scientific discussions regarding whether glucagon‐like peptide‐1 receptor agonists (GLP‐1RA) might specifically induce skeletal muscle atrophy. Consequently, multiple companies have invested significantly in muscle‐preserving strategies, including the acquisition of drugs targeting the myostatin pathway.

Despite this growing interest, long‐term human data on muscle mass alterations under semaglutide or tirzepatide therapy remain sparse. One of the few publicly shared findings stems from Versanis Bio Inc. (now a subsidiary of Eli Lilly), presented at Obesity Week 2023 [3]. In that study, mice receiving a high‐fat diet and treated with semaglutide or tirzepatide for 3 weeks exhibited approximately 10% lean body mass loss, equivalent to roughly 42% of the total weight reduction, yet skeletal muscle mass declined by only ~4%. A separate study by Paul Titchenell's lab corroborated these observations, reporting a 10% decrease in lean body mass in mice treated with a lower dose of semaglutide, although 7–10% of that was attributed to muscle mass [4]. The remainder likely included reductions in other lean compartments such as the liver or kidneys, which contract rapidly with caloric restriction and incretin therapy [5]. The absence of a pair‐fed control, however, limited direct comparisons with physiological weight loss.

Dr. Langer also described his own work, where high‐fat diet fed mice were treated with varying regimens—including a GLP‐1 RA, glucagon monotherapy, a triple agonist and a pair‐fed group (normalized to the food intake of GLP‐1RA). Body weight dropped significantly in all groups, with lean body mass accounting for 25%–50% of the total weight loss. Importantly, reductions in lean body mass were more pronounced with simple calorie restriction (i.e., pair‐feeding) than with GLP‐1RA treatment. Irrespective of physiological and pharmacological weight loss, actual skeletal muscle declines hovered only around 10%, and absolute grip strength measurements remained largely unaffected. When normalized to body weight, both muscle mass and grip strength improved for most groups. Importantly, both types of weight loss reduced liver mass by ~30%–50%. In a second study employing a triple agonist, Dr. Langer noted concurrent reductions in fat and lean body mass, whilst relative grip strength improved. Intriguingly, animals with the greatest lean body mass loss performed best on treadmill running tests, supporting the hypothesis that non‐contractile compartments may be disproportionately responsible for overall ‘lean’ tissue depletion. Despite potentially frail appearances, these mice realized substantial functional gains.

From these findings, Langer inferred that a clear discrepancy exists between reductions in lean body mass and true skeletal muscle mass. Although semaglutide and similar agents may cause significant lean tissue loss, the liver and other lean compartments may decrease at a faster rate than skeletal muscle, at least in certain animal models. Additionally, functional measures, such as grip strength, can remain stable or even trend upward when expressed relative to body weight. Dr. Langer concluded by remarking that the phenomenon warrants further long‐term clinical investigation.

Continuing, Dr. Lars Johansson (Antaros Medical, Plano, TX, USA) presented on the practical differences between lean body mass (also known as fat‐free mass) and actual muscle volume, as measured by modern imaging techniques in clinical trials. According to Johansson, the fundamental contrast lies between dual‐energy X‐ray absorptiometry (DXA) and magnetic resonance imaging (MRI), where recent studies frequently highlight that MRI and CT scans can attribute approximately 50% of adipose tissue volume to what is technically referred to as ‘lean body mass’ [1]. This distinction, he explained, becomes highly relevant when quantifying changes in adipose tissue via cross‐sectional imaging. Any adipose reduction may simultaneously register as a drop in lean body mass if proper corrections are not applied. He cited a recently published paper by Tinsley and Heymsfield in the Journal of the Endocrine Society (2024) [6] and a 2013 publication comparing whole‐body MRI and DXA [7], both of which underscore the disparities that arise, particularly in individuals with higher fat mass.

Johansson described how DXA conventionally measures fat‐free mass and fat mass, providing regional assessments such as appendicular fat‐free mass, whereas MRI can yield detailed volume measurements of not only muscle tissue but also organs like the liver, kidneys and lungs, as well as multiple adipose tissue compartments (for instance, visceral, subcutaneous, renal sinus, intramuscular, perirenal and epicardial). He emphasized that MRI can isolate so‐called ‘adipose tissue‐free muscle volumes’, sometimes referred to as ‘contractile muscle volume’. However, he cautioned that this term might be slightly misleading, as the measured volume may include fibrosis or other components beyond purely contractile fibres. Johansson suggested ‘lean muscle volume’ as more precise terminology, because it accounts for intramyocellular fat (IMCL). MRI can further differentiate intramuscular adipose tissue (IMAT) from intermuscular adipose tissue, providing a clearer lens into true muscle composition.

At the whole‐body level, modern MRI techniques can delineate subcutaneous fat, visceral fat and adipose tissue‐free volume (including hepatic or renal fat). This perspective becomes critical when evaluating reductions in these non‐contractile compartments, which may influence overall lean tissue volume changes but not reflect true muscle mass loss. Johansson noted that advanced AI‐based segmentation now automates the mapping of individual muscles, adipose tissue and even bones. Such automation facilitates the accurate calculation of muscle volumes and fat content across specific muscles, which is invaluable since different muscle types (e.g., fast‐twitch vs. slow‐twitch) may respond variably to certain therapies.

Reflecting on earlier experiences at AstraZeneca, Johansson recalled using whole‐body MRI to study peroxisome proliferator–activated receptors (PPARs) in patients undergoing bariatric surgery. Over the course of a year, researchers documented dramatic reductions in BMI and subcutaneous adipose tissue, along with improvements in insulin sensitivity and metabolic parameters. Interestingly, total muscle fat tended to decrease under significant caloric restriction; however, intramyocellular fat (IMCL) paradoxically rose, highlighting the intricate interplay between adipose distribution and metabolic states. Lastly, Johansson pointed out that functional evaluations, such as hyperinsulinemic‐euglycemic clamps with labelled glucose injections, can complement imaging by offering deeper insights into muscle metabolism.

### 2.1 Loss in Fat‐Free Mass: Patterns, Predictors and Gaps in Incretin Trials

Prof. Steven Heymsfield (Pennington Biomedical Research Center, Baton Rouge, LA, USA) delivered a detailed presentation focused on the effects of incretin therapies on skeletal muscle mass, particularly examining available trial data and the current knowledge gaps. He began by posing two fundamental questions: how much muscle mass is typically lost during calorie‐restricted weight loss, and how much muscle is lost when a comparable caloric deficit is achieved with the aid of an incretin? Whilst the first question can be reasonably answered, the second remains unresolved, which framed the core of his discussion.

The issue gained visibility following the publication by Jastreboff et al. in the *New England Journal of Medicine* [9], which presented weight loss data indicating that, for a 100‐kg individual losing 20 kg (i.e., 20% body weight), approximately 25% of that loss (5 kg) comes from lean body mass, with about half (2–2.5 kg) attributable to muscle mass. Although these figures are broad estimates, they offer a foundational context for interpreting subsequent findings.

Heymsfield referenced a review article by Bob Dubin, to which he contributed, highlighting the wide variability in fat‐free mass loss across studies, ranging from 10% to 40% [10]. Jennifer Linge's related work further confirmed a correlation between weight loss and lean body mass loss, typically along a regression line of approximately 50% [11]. Additional studies, including those on bariatric surgery patients, have revealed similarly significant reductions in lean body mass [12]. The consistent presence of lean body mass loss in weight reduction interventions suggests a fundamental physiological response.

However, Heymsfield emphasized common key limitations across these studies. These include considerable variation in treatment duration, lack of sex‐specific analyses (often pooling male and female data), inconsistent definitions of ‘lean body mass’, and a frequent absence of predictive modelling for expected changes during caloric restriction. From studies of calorie restriction, he noted that early fat‐free mass loss is typically steep but plateaus between 10 and 20 weeks. Animal models illustrate that muscle mass is often preserved relative to other lean tissues, such as the liver, which tends to atrophy more rapidly. This variability reflects the heterogeneous nature of lean body mass. In studies examining cardiac atrophy during starvation, heart mass loss was shown to scale with body weight reduction, paralleling that of skeletal muscle. Similarly, in chronic caloric restriction, heart mass loss was in proportion to muscle loss.

A 9‐ to 10‐week study of hypocaloric dieting revealed an average body weight loss of 10% without substantial bone loss in the short term. DEXA‐based body composition analysis indicated that fat loss occurred faster than lean soft tissue loss, and that men experienced more significant lean body mass reductions than women. On average, lean body mass accounted for 33% of total weight loss in men and 25% in women, supporting a clear gender difference in the physiological response to weight loss.

Heymsfield clarified several key definitions in the context of body composition [13]:

- Fat‐free mass (FFM): Includes bone and lean soft tissue.
- Lean soft tissue (LST): Excludes bone.

Approximately half of LST is found in the appendicular region, with roughly half of that comprised of fat‐free skeletal muscle. The inconsistent and overlapping use of terms like FFM, lean body mass and LST often leads to confusion in interpreting research findings. He also noted that as body weight increases, muscle mass typically follows in a roughly linear relationship. Conversely, during weight loss, muscle atrophy is commonly observed. Cross‐sectional studies indicate that men lose around 2–2.5 kg of muscle for every 10 kg of body weight lost, whilst women lose approximately 1–1.5 kg [14]. These findings align with traditional models of weight loss distribution: roughly 75% from fat mass and 25% from lean body mass, with gender‐based variation [15].

Prediction models allow researchers to estimate expected changes in fat and lean compartments during weight loss. Regression lines have been established for LST and fat mass, allowing for relatively accurate forecasts of changes in body composition. However, it is unclear whether these trends hold when GLP‐1RA, such as semaglutide or tirzepatide, are introduced. Trials like STEP 1 and SURMOUNT 1 report changes in lean body mass [1, 9], but often lack details on measurement specificity. Available data suggest that what was measured was lean soft tissue minus bone, but more clarity is needed.

Whilst skeletal muscle represents the largest component of lean body mass, other organs such as the heart and liver also contribute significantly to overall metabolism and physical function. For example, a 10%–20% reduction in cardiac mass could carry more significant physiological consequences than a similar reduction in skeletal muscle mass [16]. Heymsfield concluded by emphasizing the importance of obtaining precise, tissue‐specific body composition measurements to accurately assess the metabolic and clinical impact of weight loss therapies, particularly in the context of incretin use.

### 2.2 Weight Regain After Intervention Withdrawal

How does body composition change after discontinuation of incretin therapy? Prof. W. Timothy Garvey (University of Alabama at Birmingham, Birmingham, AL, USA) transitioned to this topic in his presentation. He began by noting that, although powerful GLP‐1‐based anorexigenic agents such as semaglutide and tirzepatide can induce substantial weight loss, real‐world data show that fewer than 50% of patients remain on these medications after 1 year. When therapy is stopped, weight regain is frequently observed. This raised the central question of his talk: Does the regained weight have the same composition as the lost weight?

Garvey framed this issue within the broader context of ‘yo‐yo dieting’, where body weight repeatedly cycles through loss and regain phases [17, 18]. It is commonly assumed that repeated weight cycling leads to a disproportionate regain of fat mass at the expense of lean tissue. This belief is partly based on older data, including the Minnesota Starvation Experiment led by Ancel Keys. In that study, male volunteers underwent a period of semi‐starvation followed by refeeding. Both lean and fat mass declined during starvation; however, whilst fat‐free mass generally returned to baseline after refeeding, some participants experienced an overshoot in fat mass. This overshoot appeared to be more common among individuals with lower baseline fat stores, whereas those with greater adiposity did not experience this effect.

Garvey also referenced cachexia‐related conditions as further examples contributing to the perception that weight regain favours fat accumulation. But whether this also applies to patients with obesity who discontinue incretin therapy remains uncertain. He drew attention to data from *The Biggest Loser*, where participants lost approximately 60 kg over 30 weeks and regained 40 kg within 6 years [19]. Importantly, no evidence of preferential fat regain was observed; instead, both fat and lean body mass appeared to follow the same proportional trajectory during both phases.

He then cited a systematic review by Sanaya et al., which examined body composition during weight cycling [20]. Of the 20 studies analysed, 15 reported no preferential regain of fat mass, and none of the 18 studies that reported changes in lean body mass found a significant decrease in lean body mass during weight regain. These findings challenge the prevailing assumption that weight regain inevitably leads to a deterioration in body composition. One notable exception involved postmenopausal women. A study by Beavers et al. found that during a five‐month weight loss period, 67% of the weight lost was fat and 33% was lean body mass [21]. At both 6 and 12 months following weight regain, approximately 80% of the regained weight was fat and 20% was muscle. The authors interpreted this as evidence of preferential fat regain. However, Prof. Garvey noted that the lean‐to‐fat mass ratio remained higher than baseline even at 12 months, and the trajectories of lean versus fat mass during loss and regain did not differ significantly, suggesting the interpretation may be more nuanced.

In the course of his presentation, he emphasized that older individuals, particularly those over 70 years of age, face greater difficulty regenerating muscle after weight loss. In these populations, weight regain is more likely to consist predominantly of fat, increasing health risks associated with sarcopenia and frailty. He referenced work by Steve Kuczewski, which showed that in older adults, nearly 50% of the weight lost during intentional dieting was lean body mass, yet virtually none of the regained weight was lean. These findings underscore the particular vulnerability of older patients.

Garvey transitioned into discussing incretin‐specific mechanisms. Despite claims at various public presentations that post‐incretin weight regain consists of 80% to 100% fat mass, no published data currently confirm this. He questioned the empirical basis of these assertions and stressed the need for caution. Muscle regeneration may lag behind weight regain, but this does not necessarily imply a pathological shift in body composition. He argued that, rather than placing blame on incretin therapies, these treatments appear to have a similar effect to conventional dieting. The weight regain pattern observed after stopping therapy closely resembles that seen after voluntary weight loss interventions. Whilst incretins may temporarily shift the body's weight ‘set point’, the idea of a rebound into a ‘starvation mode’ that drives fat regain lacks scientific support.

Finally, Prof. Garvey addressed the terminology used in this field. He cautioned against using the term ‘incretin’ too broadly, as it encompasses multiple hormones with distinct biological effects—namely, GLP‐1, GIP and glucagon. Lumping them together, he argued, is as imprecise as referring to anabolic, glucocorticoid and mineralocorticoid steroids simply as ‘steroids’. Such distinctions are critical for accurately interpreting both mechanisms and outcomes in metabolic therapies.

### 2.3 Muscle Volume, Composition and Confounders

Dr. Jennifer Linge (AMRA Medical, Linköping, Sweden) then presented on the challenges of analysing muscle health and body composition in individuals with obesity, a population quite different from those with cachexia or uremic conditions traditionally studied in sarcopenia research. She began by acknowledging that the field has historically relied on assessments of mobility, physical performance and muscle strength. However, these functional tests are highly influenced by numerous confounding factors, including body weight, physical fitness, pain, motivation, muscle mass, and, in particular, muscle composition. For individuals with obesity, weight loss can lead to apparent improvements in physical tests; however, these do not necessarily reflect preserved muscle mass or composition and may mask longer‐term risks.

**FIGURE 1:**
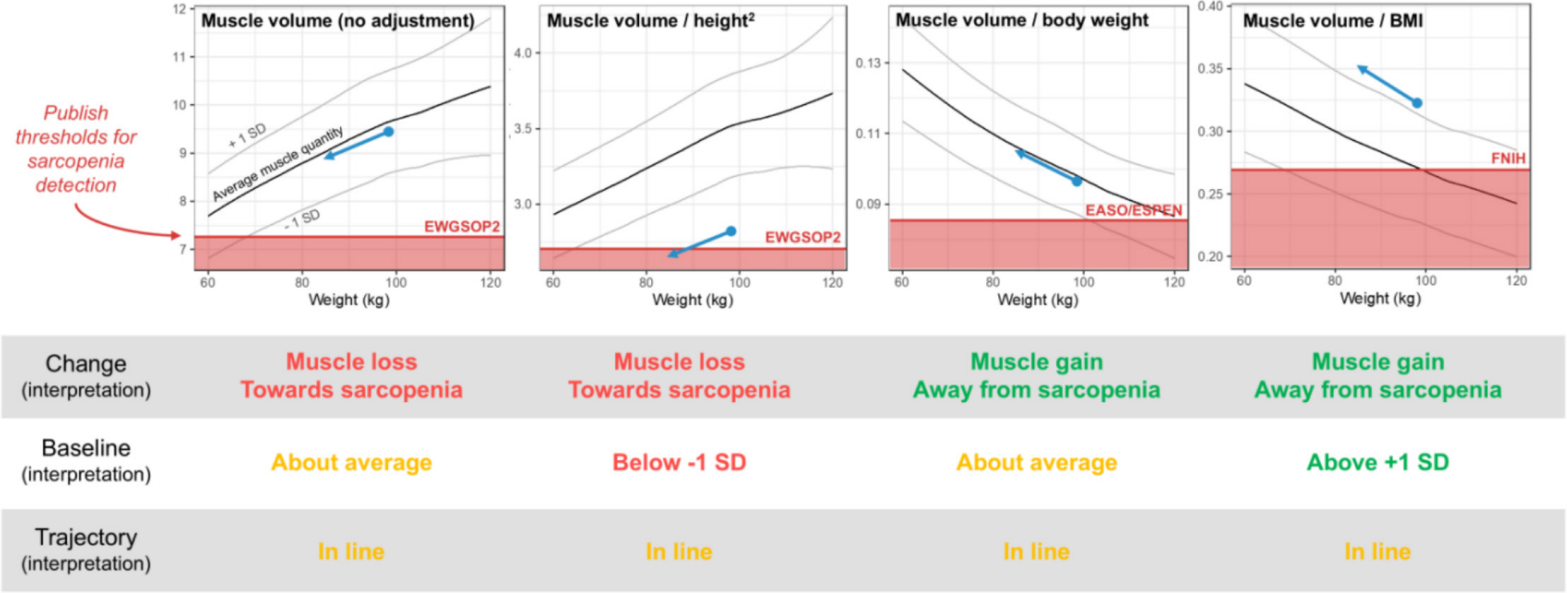
Interpretation of muscle changes during weight loss.

Dr. Linge emphasized that body weight itself is a key confounder in evaluating muscle mass. As previously noted by Heymsfield, individuals with obesity often exhibit greater absolute muscle mass, and some degree of muscle loss is anticipated during weight reduction. Nonetheless, defining what constitutes a ‘normal’ or acceptable degree of muscle loss remains a significant challenge. Moreover, long‐term health outcomes are not solely dependent on the quantity of muscle but also on its quality and composition. Traditional normalization techniques in sarcopenia research—adjusting muscle volume for height squared, weight or BMI—fail to remove the correlation with body weight adequately. Linge illustrated this using a series of scatter plots where muscle volume remains correlated with weight regardless of the adjustment method (Figure 1). This issue is compounded by heterogeneous sarcopenia thresholds proposed by various European groups, further complicating standardization.

To demonstrate this complexity, she presented the case of a woman undergoing GLP‐1 receptor agonist therapy combined with lifestyle intervention [22]. The patient lost 13.7% of her total body weight and 5.7% of her thigh muscle volume. Depending on the chosen adjustment method, her data could be interpreted as either progression towards sarcopenia or improvement away from it. Even her baseline data yielded conflicting interpretations. The only consistent pattern was that her muscle volume changes paralleled changes in body weight, reinforcing the need for more robust evaluative methods.

In response to this issue, she introduced the ‘muscle volume Z‐score’, developed to be independent of height, weight and BMI [23]. This metric compares an individual's muscle volume to that of a matched reference population. In cases where absolute muscle volume remains stable during weight loss, an increase in the Z‐score suggests improvement in relative muscle health. Conversely, if muscle volume declines in proportion to weight loss, the Z‐score remains stable. This approach enables more accurate interpretation of changes in muscle volume relative to expected trends. She highlighted the utility of MRI and CT imaging in assessing muscle composition, particularly intramuscular fat infiltration, which correlates more strongly with clinical outcomes than raw muscle volume. In the Study of Muscle, Mobility and Aging (SOMMA), raw muscle volume showed modest associations with strength and mobility tests. However, when using the muscle volume Z‐score, the strength of these correlations improved significantly. Notably, muscle fat infiltration showed the strongest association with declines in physical function.

Dr. Linge also presented data from the UK Biobank, which included 40 000 participants. Individuals with normal muscle composition had the lowest all‐cause mortality, followed sequentially by those with low muscle volume Z‐scores, those with high muscle fat infiltration, and those with adverse muscle composition [24]. ‘Adverse muscle composition’ refers to the combined presence of both low muscle volume and high intramuscular fat, and is associated with poor muscle function [25]. The latter group had a hazard ratio of 3.7 for mortality, and these trends held true across subgroups including individuals with obesity, metabolic dysfunction‐associated liver disease (MAFLD), and chronic kidney disease [25].

She concluded by emphasizing the need for accurate and weight‐independent methods of evaluating muscle mass and quality. Tools like the muscle volume Z‐score and detailed imaging can improve clinical and regulatory decision‐making in assessing therapies such as GLP‐1RA.

### 2.4 Imaging Body Composition: The Challenge of Standardization

As the final presenter of this session, Dr. Edvin Johansson (Antaros Medical, Plano, TX, USA) provided a technical perspective on imaging methodologies for body composition assessment beyond DEXA. His talk highlighted the importance—and the ongoing difficulties—in achieving standardization across imaging endpoints, particularly in large‐scale clinical trials involving patients with obesity.

Dr. Johansson began by reiterating that whilst the pros and cons of methods such as CT, MRI and DXA are well established, DXA is the only technique that has reached a high level of standardization [26]. MRI, however, is rapidly evolving, and platforms such as AMRA have made significant contributions to the field. Still, the lack of universal definitions and imaging protocols continues to hinder meaningful comparisons across trials [27]. He acknowledged that reviewing published data is often frustrating because the imaging methodology is rarely described in sufficient detail. This lack of clarity makes it difficult to determine how measurements were obtained, particularly for adipose tissues. For example, whole‐body imaging can vary from true head‐to‐toe scans to partial protocols, such as T9 to knee, or region‐specific approaches, like scanning the abdomen or thighs using either slab or single‐slice techniques. Each of these variations can significantly impact measured values, particularly for fat compartments. Patient size further complicates the issue. In MRI and CT, large patients may not fit entirely within the scanner field, resulting in missing data for the arms or portions of subcutaneous fat. Researchers must then decide whether to exclude these data, interpolate missing values or report the omission explicitly. For instance, subcutaneous adipose tissue is frequently truncated when the scanning field limits coverage to 45 cm, leaving substantial fat deposits unmeasured. Visceral adipose tissue (VAT) poses even more definitional challenges. Despite being measured in over 50 trials, there remains no consensus on what VAT encompasses. It may encompass peritoneal, retroperitoneal, epicardial and omental fat, but the choice often depends on the scan slice's position and is rarely described in methods sections. Similarly, whether single‐slice, slab or volumetric imaging is used has a significant impact on which fat compartments are captured.

Dr. Johansson illustrated this complexity with an anatomical image showing omental fat, para‐renal fat, peri‐renal fat and epicardial fat. In many studies, these compartments are often lumped together as VAT, although they differ biologically and may have unique clinical implications. For example, epicardial fat requires a dedicated cardiac imaging protocol, whilst peri‐renal fat includes a higher proportion of brown adipocytes compared to para‐renal fat. Such biological and imaging‐based distinctions are seldom acknowledged in study reports.

Renal fat is a relatively new focus in obesity trials, yet even here, standardization is lacking. Renal parenchymal fat is typically below detection thresholds, whilst peri‐renal and renal sinus fat are more accessible. However, no clear anatomical consensus exists on where to draw the boundaries, particularly in noisy images where distinguishing the fascia between compartments is challenging. This ambiguity introduces variability that undermines cross‐study comparison.

In his concluding remarks, Dr. Johansson emphasized that standardization is still missing for many organs and fat depots assessed via MRI or CT. Whether using slices, slabs or full‐body imaging, each approach yields different anatomical coverage and, thus, different endpoint values. Whilst AI‐based segmentation tools offer promise in automating and expanding tissue compartment analysis, their utility hinges on the adoption of clear, universally accepted definitions and imaging protocols.

### 2.5 Discussion

Following the presentations, a wide‐ranging discussion ensued, focusing on the implications of lean body mass loss during weight reduction, especially in the context of incretin‐based therapies. Prof. Vickie Baracos initiated the discussion by posing a fundamental question: Does muscle loss in obese individuals undergoing weight reduction constitute a medical emergency, an urgent concern or perhaps a benign physiological adjustment? Drawing from her own calculations, she illustrated how morbid obesity requires considerable muscle mass to maintain mobility. Therefore, she hypothesized that part of the observed muscle loss during weight reduction could simply reflect the body adapting to a decreased mechanical load. However, she emphasized that this adaptive capacity may be significantly impaired in older individuals, particularly those over 75 or 80 years of age. Baracos elaborated that caloric restriction suppresses muscle protein synthesis, and aging further exacerbates this decline due to diminished dietary protein intake, hormonal deficits (e.g., testosterone, growth hormone and IGF‐1) and decreased physical activity. She emphasized that in older adults, regaining lost muscle mass is nearly impossible unless special interventions, such as resistance training, are implemented. She also noted the limited long‐term data on incretin‐based therapies and underscored the need for a better understanding of their effects on patient‐reported outcomes and physical function. She referenced emerging evidence suggesting that preserving muscle during GLP‐1RA therapy could amplify fat loss and metabolic benefits, although such outcomes remain to be confirmed in future studies [28].

Whereas some participants raised the counterpoint that not all lean body mass should be prioritized equally. For example, in animal models, muscle mass decreased by about 10%, whilst liver mass declined by 50%–60%. These observations raise the question of whether changes in lean body mass should be prioritized over other factors in evaluating therapeutic outcomes.

Baracos cautioned that repeated weight cycling could lead to progressive sarcopenia, especially in aging populations. She framed two major concerns: safety and efficacy. Whilst trials such as SELECT have not revealed severe safety signals, the unique vulnerabilities of older patients warrant targeted research. On the efficacy front, combining weight loss agents with muscle‐preserving compounds might enhance both metabolic outcomes and patient function. Despite some functional gains observed in trials such as STEP‐1 and SURMOUNT‐1 [1, 9], particularly in patient‐reported outcomes, it remains uncertain whether these improvements are clinically meaningful. To demonstrate harm or benefit more conclusively, future studies should target older, at‐risk populations or explore the potential for enhanced efficacy through combined therapies. She concluded by stating that the ultimate goal is not just to ensure the safety of these drugs but to make them significantly more effective, whether by reducing mortality, improving physical function or addressing other patient‐centred outcomes.

Dr. Scott Harris (Altimmune Inc., Gaithersburg, MD, USA) then noted that several trials, including SELECT, reported up to a 40% loss of lean body mass with semaglutide, a finding with potential clinical relevance. He referenced the Look AHEAD trial and the labelling of semaglutide, both of which noted increased fracture risks in older adults. Whilst causality remains unclear, Harris argued that such morbidity should not be dismissed, particularly when lean body mass loss may be implicated. He urged the field to start with the assumption that lean body mass loss matters and explore whether dual or triple agonists, or adjunctive therapies, could mitigate these effects.

In the final portion of the discussion, attention turned to regulatory perspectives on the long‐term implications of powerful weight‐loss therapies (Table 1). A participant raised a question directed to regulators regarding diseases with relatively well‐understood progression, such as chronic kidney disease (CKD) and chronic heart failure. Specifically, they inquired about how regulatory agencies assess the trial timelines for medications intended for chronic or intermittent use, particularly when such therapies may be used repeatedly for lifestyle or aesthetic purposes—for example, seasonal weight loss regimens. They questioned whether the evaluation of long‐term effects in these contexts should be limited to post‐marketing surveillance or warrant dedicated pre‐approval studies.

**TABLE 1:** Summary of important regulatory discussion points.

Important regulatory discussion points
1 It is uncertain what clinically meaningful changes in PROs, physical function and/or morbidity/mortality outcomes could support regulatory approval.
2 A reasonable amount of safety data needs to be provided considering that the treatment indication sought after likely includes many millions of patients.
3 If PROs are used, they need to be validated instruments relevant for the patient population they are used in.
4 It appears that incremental weight loss could support regulatory approval [8].
5 Higher amounts of fat mass loss or a gain in muscle mass appear not to be sufficient for regulatory approval, but data on this needs to be available to understand the impact of the therapy and they can support a mode of action discussion.
6 General physical activity data may be of interest, if the tool is providing valid data.

Abbreviation: PROs = patient‐reported outcomes.

A regulatory representative acknowledged that whilst incretin‐based treatments are approved as chronic therapies, their real‐world usage patterns vary significantly. In many cases, patients are using them intermittently rather than continuously. Whether such use raises specific safety concerns remains uncertain and will likely only be clarified through ongoing post‐marketing surveillance. Without trials explicitly designed to evaluate intermittent or aesthetic use, regulators must rely on real‐world data to assess these outcomes. The representative added that bariatric surgery, which often results in even more substantial and durable weight loss, has not led to significant long‐term impairments in physical activity in the general population. However, they cautioned that certain subgroups, such as older adults, may still be at increased risk and require further investigation.

Subsequent comments from a participant highlighted the importance of considering age when evaluating absolute risk and treatment efficacy. For example, obesity trials typically enroll participants with an average age of around 46 years, whilst liver trials skew slightly older at 55–56. Cardiovascular outcome trials, such as SELECT, typically average 61 years, and trials for chronic kidney disease (CKD) or heart failure (e.g., DAPA‐CKD and EMPA‐CKD) often include patients aged 63 to 68. These differences in baseline age alter the absolute risk landscape, which must be considered when interpreting outcomes across diverse clinical populations.

Dr. David J. Glass (Regeneron Pharmaceuticals, Tarrytown, NY, USA) then raised an important regulatory consideration regarding younger patients. Whilst muscle loss in this demographic may not pose an immediate safety risk, preserving muscle might confer secondary benefits, such as enabling more significant fat loss or improving long‐term metabolic outcomes. He posed the question of whether preserving muscle—even at the expense of reduced total weight loss—would complicate regulatory approval, given that muscle gain could offset fat loss in total weight calculations. He also queried whether regulators distinguish between fat and overall weight in their efficacy evaluations.

In response, a regulator clarified that although body composition scans are not standard in clinical practice, obesity is formally defined as a disease of excess adiposity. Regulatory guidance thus emphasizes the importance of demonstrating that weight loss is primarily attributable to fat mass reduction. Whilst no precise threshold for ‘primarily’ is mandated, a drug that produced substantial lean body mass loss without adequate fat mass reduction would likely face serious hurdles in the approval process.

Dr. Aminah Jatoi (Mayo Clinic, Rochester, MN, USA) noted that her understanding was that only about 7 years of post‐marketing data currently exists for GLP‐1 RA. She expressed concern that this limited timeframe may not be sufficient to fully evaluate long‐term safety and efficacy profiles.

Prof. Heymsfield further expanded on a mechanistic point related to body composition. He asked whether the relationship between fat and muscle might be mediated by shared metabolic mechanisms and whether co‐administration of anabolic agents could enhance fat loss as effectively as current monotherapies. Another participant responded by highlighting that muscle is metabolically active and competes for caloric resources. Increasing muscle protein synthesis elevates resting energy expenditure, thereby facilitating fat loss. One major cause of reduced metabolic rate during dieting is the suppression of muscle protein synthesis; thus, maintaining muscle through anabolic support may counteract this drop and lead to better fat loss outcomes.

## 3 Treatment Approaches to Address Muscle Wasting in the Context of Obesity Therapy and Regulatory Issues

### 3.1 Bimagrumab: Dual Targeting of Muscle Growth and Fat Loss

The opening presentation of this session was delivered by Dr. Kenneth Attie (Eli Lilly/Versanis Bio, NY/IN, USA), who presented a detailed overview of bimagrumab, a monoclonal antibody targeting the activin type 2 receptor, originally developed by Novartis. His presentation focused on the mechanistic rationale and clinical data supporting its use in treating obesity and muscle loss.

Bimagrumab operates by inhibiting signalling through type 2 activin receptors, which are negative regulators of muscle growth. By blocking this pathway, the drug promotes muscle protein synthesis whilst inhibiting muscle protein degradation. Dr. Attie highlighted that although the signalling components differ between muscle and adipose tissue, with ALK4 serving as the type 1 receptor in muscle and ALK7 in adipose tissue, the shared type 2 receptor enables bimagrumab to exert effects in both tissues. In adipose tissue, the drug stimulates fat mobilization and lipolysis, raising the question of whether its fat‐reducing effects stem solely from increased muscle mass or also from direct actions on adipocytes. The dual‐site activity of bimagrumab may offer unique advantages in managing obesity‐related conditions.

Dr. Attie then discussed clinical data from a study of individuals with obesity and type 2 diabetes, conducted by Prof. Steven Heymsfield and the Novartis team [29]. In this study, participants experienced a 4% increase in lean body mass as measured by DEXA and a comparable increase in appendicular lean body mass. MRI data further showed a 6% increase in paravertebral muscle mass in a subset of patients, suggesting that DEXA may underestimate true skeletal muscle gains due to its inability to differentiate between contractile and non‐contractile lean tissues. Fat mass was reduced by approximately 20%, a level comparable to semaglutide, whilst overall weight loss was only 7%. This discrepancy was attributed to the concurrent gain in lean body mass, suggesting that total body weight may not fully capture the therapeutic impact of bimagrumab. Notably, reductions in visceral adipose tissue and hepatic fat fraction were also observed, whilst trends towards decreased intramyocellular and intermuscular fat did not reach statistical significance. These compositional changes correlated with a 1% reduction in haemoglobin A1c, underscoring the metabolic benefits of the treatment.

Dr. Attie then described the ongoing BELIEVE study, initiated prior to Versanis' acquisition by Eli Lilly [NCT05616013]. This multinational, factorial‐design trial includes approximately 500 participants and is evaluating the effects of bimagrumab alone and in combination with semaglutide. Participants receive one of two doses of each drug for 72 weeks, followed by a 32‐week withdrawal period and final follow‐up. Body composition is assessed using DEXA at weeks 12, 24, 48, 72 and 96, allowing for detailed evaluation of both treatment and post‐treatment effects.

In summarizing the key findings, Dr. Attie emphasized that bimagrumab achieves a meaningful reduction in fat mass without the lean body mass losses commonly associated with other weight‐loss drugs. Compared to agents like semaglutide and tirzepatide (as shown in STEP 1 and SURMOUNT‐1) [1, 9], bimagrumab demonstrates a more favourable ratio of fat‐to‐lean mass loss. However, he acknowledged that preserving lean body mass whilst achieving meaningful weight loss necessitates more significant fat loss overall to match the total weight reduction seen with other agents. This presents both an opportunity and a challenge for the development of future obesity therapies.

He concluded by urging the field to reconsider current outcome metrics in the development of obesity drugs. By shifting focus from total weight loss to changes in body composition, particularly fat loss versus lean body mass preservation, clinical benefits can be better contextualized and optimized.

### 3.2 Pemvidutide: A Dual Agonist Balancing Weight Loss and Lean Body Mass Preservation

Dr. Scott Harris followed with a presentation on pemvidutide, a dual receptor agonist targeting both GLP‐1 and glucagon receptors. Reflecting on his career trajectory, Dr. Harris noted a shift from earlier research aimed at increasing weight and lean body mass, such as work with a ghrelin agonist in critically ill patients, to his current focus on promoting weight loss whilst preserving body composition. He referenced early collaboration with Bill Evans using the D3 creatine dilution method to measure muscle mass, which laid the groundwork for his enduring interest in lean body mass metrics.

The focal point of his presentation was the Momentum Trial, a Phase 2 clinical study evaluating pemvidutide in 391 overweight or obese individuals over a 48‐week period. The trial featured three dosing arms (1.2, 1.8, and 2.4 mg) and a placebo group. Notably, unlike other incretin‐based therapies which require gradual dose escalation over 20 to 32 weeks to ensure tolerability, pemvidutide was administered at therapeutic doses from the start, with the highest dose reached by week four. All participants received standard lifestyle interventions, and body composition was assessed in 67 subjects via MRI.

Key results:

- Average weight reduction at 48 weeks: 15.6%
- Lean body mass loss ratio: 21.9% of total weight lost

By comparison: ~40% with semaglutide, ~25% with diet and exercise

- Regression analysis (rather than mean group comparison) showed consistent preservation across individuals (coefficient of variation: 0.663)
- Age‐stratified data indicated that lean body mass preservation was maintained even in participants over 60 years old, with no clinically significant deterioration

Dr. Harris emphasized pemvidutide's dual mechanism, pairing GLP‐1's effects on appetite suppression and glucose regulation with glucagon's potent influence on lipid metabolism. Glucagon shifts energy utilization from carbohydrates to fats, leading to reductions in liver fat, serum lipids and overall adiposity. These metabolic effects were proposed as the basis for pemvidutide's apparent capacity to preserve lean body mass during weight reduction. Dr. Harris also underscored that pemvidutide's ability to preserve lean body mass may make it particularly valuable in older adults, who are at a higher risk of sarcopenia and fractures during weight loss. He concluded that the 21.9% lean body mass loss ratio may represent a class‐leading outcome among dual receptor agonists, with efficacy potentially superior to that of lifestyle intervention alone. The findings, originally presented at the European Diabetes Meeting, offer promising directions for balancing weight reduction with skeletal muscle integrity in future metabolic therapies.

### 3.3 Preserving Strength: Enobosarm as an Adjunct to Incretin‐Based Weight Loss

Dr. Mitchell Steiner (Veru Inc., Miami, FL, USA) delivered the third presentation, highlighting the rationale behind combining GLP‐1RA‐based therapies with anabolic agents to improve weight loss outcomes. He began by reflecting on his nearly two decades of experience in the field, initially focusing on frailty. Drawing from that foundation, he emphasized that despite advances in weight loss therapies, a core challenge remains: how to assess meaningful endpoints. His presentation centred on two key points: the role of lean body mass and muscle in GLP‐1 treatment, and how preserving lean body mass can reshape the patient's weight loss journey. He also shared insights from discussions with the FDA, which have been broadly positive but still marked by important uncertainties.

To set the stage, Steiner introduced Enobosarm, Veru's investigational selective androgen receptor modulator (SARM) [30, 31]. Originally developed to emulate the anabolic effects of testosterone whilst minimizing androgenic side effects, enobosarm reduces fat mass, increases muscle mass and improves function [32, 33]. Critically, it is not aromatized to oestrogen or converted to dihydrotestosterone (DHT), thus avoiding risks such as prostate hypertrophy or virilization. This makes it especially relevant for women, who constitute approximately 70% of GLP‐1 users. Enobosarm has been tested in over 2000 individuals across 27 clinical trials and has demonstrated a strong safety profile, including no evidence of drug‐induced liver injury.

Dr. Steiner then discussed the concept of the ‘weight loss journey’, emphasizing that muscle is not only essential for physical function but also plays a metabolic role in maintaining insulin sensitivity and regulating energy balance. GLP‐1RA, whilst effective in reducing appetite and promoting fat loss, also results in absolute reductions in lean body mass. This can activate physiological compensatory mechanisms, such as myokine‐mediated signalling to the brain, potentially increasing hunger. Steiner proposed that co‐administering a muscle‐preserving agent, such as enobosarm, could mitigate these effects, improve metabolic resilience and support more favourable long‐term outcomes.

He further addressed an emerging regulatory challenge: determining the clinical significance of incremental weight loss improvements. Historically, a 5% reduction in total body weight has served as a benchmark for the approval of anti‐obesity medications, and combining drugs to meet or exceed this threshold has been generally acceptable. However, as newer therapies now routinely achieve weight reductions well above 10%, the relevance of additional reductions becomes more nuanced. Steiner noted that it remains unclear how much additional benefit is conveyed by each incremental gain and what constitutes a meaningful improvement from the patient's perspective. He emphasized that new data will be crucial in informing both regulatory and clinical decisions in this evolving landscape.

Veru's ongoing Phase 2b trial focuses on this question. Led by Prof. Steven Heymsfield, the trial includes 165 overweight or obese participants initiating GLP‐1RA therapy for the first time [NCT06282458]. It evaluates semaglutide alone versus semaglutide in combination with 3 mg or 6 mg of enobosarm over a 16‐week period. The primary endpoint is the change in lean body mass. Secondary endpoints include total body weight, fat mass, stair climb performance and haemoglobin A1c levels. The trial aims to demonstrate that enobosarm preserves muscle whilst enhancing fat loss and improving physical performance, which may support greater post‐treatment weight stability.

Dr. Steiner emphasized that physical function, particularly as measured by stair climb tests, is a meaningful clinical endpoint. These tests mirror everyday activities, such as getting out of a chair or ascending stairs, and provide a reliable metric of patient capability. Previous trials, including studies on cancer cachexia, have shown that enobosarm not only preserves lean body mass but also improves functional performance, with observed gains of up to 15%, a magnitude that he noted is clinically meaningful.

In conclusion, Steiner reiterated the importance of shifting the focus from weight loss alone to improvements in body composition and physical function. The ongoing trial aims to demonstrate that combining enobosarm with a GLP‐1RA can preserve muscle, enhance fat loss and improve physical function. This could provide a compelling case for integrating such combinations into weight management therapies, ultimately enhancing patient outcomes and quality of life. By combining anabolic agents like enobosarm with GLP‐1RA therapies, he argued, the field may advance towards more holistic obesity treatments that not only reduce fat but preserve and strengthen muscle, ultimately enhancing patient well‐being and quality of life.

### 3.4 BIO101 and GLP‐1RA Co‐Therapy

Dr. Rob van Maanen (Biophytis, France) presented on BIO101, also known as 20‐hydroxyecdysone (20E), and its potential application in combination therapies for obesity and muscle preservation. He opened by briefly recapping the mechanism already outlined by his colleague, Dr. Waly Dioh, reviewing that BIO101 acts on the protective arm of the renin‐angiotensin system [34]. This pathway promotes protein synthesis and muscle hypertrophy, a fact demonstrated in preclinical studies using aged sarcopenic mouse models. In one such study, a six‐day treatment with BIO101 increased myofiber diameter, fusion index and the number of nuclei per myotube. A 14‐week treatment normalized running velocity in treated mice, which had otherwise declined in the vehicle group [35]. In a high‐fat diet‐induced obesity mouse model, BIO101 reversed increases in epididymal fat and improved several surrogate markers of metabolic dysfunction. These promising preclinical findings laid the groundwork for clinical investigations [36].

In human studies, overweight and obese individuals participated in a structured trial consisting of a six‐week calorie‐restricted diet followed by a six‐week maintenance phase. During the diet phase, patients receiving low‐dose BIO101 showed significant reductions in visceral fat mass and adipocyte diameter compared to placebo. After the dietary intervention ended, fat mass rebounded in the placebo group but remained largely stable in the BIO101 group, suggesting a protective effect. The study also evaluated handgrip strength. Among participants who lost at least 5% of their body weight, a decrease in handgrip strength was commonly observed; however, this decline was mitigated in those receiving BIO101, although the finding did not reach formal statistical significance. A similar trend had been observed previously in studies focused on sarcopenia. He then discussed a subgroup analysis from a sarcopenia trial, in which approximately 72% of participants met the criteria for sarcopenic obesity. In this subgroup, a nominally significant improvement in walking speed was observed within the per‐protocol population.

Building on these results, Biophytis designed the ongoing OBA study, a Phase 2 proof‐of‐concept trial. This randomized, double‐blind, placebo‐controlled study includes a 21‐week treatment period, during which patients receive semaglutide as a standard background therapy. Participants are randomized to receive either 350 mg of BIO101 twice daily or a matching placebo. The trial is powered to detect changes in knee extension strength as the primary endpoint and includes 164 participants. The target population comprises overweight or obese individuals with weight‐related comorbidities, closely mirroring the demographic for which GLP‐1RA are indicated. Sites are located in both the United States and Europe, and Prof. Marc‐Andre Cornier of the University of South Carolina leads the trial. The study has received FDA clearance and IND (Investigational New Drug) approval and is currently being initiated. Key inclusion criteria include being 18 years or older, with a BMI below 40 (to allow for DXA scanning), and a willingness to maintain a protein‐enriched diet and engage in regular physical activity. Major exclusions encompass diabetes, recent significant weight changes, psychiatric safety risks and other factors that could confound results. In addition to the primary endpoint of knee extension strength, the study will assess secondary measures, including knee flexion, handgrip strength, mobility and performance metrics (e.g., 6‐min walk test, five‐times sit‐to‐stand test and stair climb test), as well as DXA‐derived body composition. Several outcomes will be normalized to individual baseline characteristics. The study will also incorporate quality‐of‐life assessments using both general (SF‐36) and obesity‐specific tools, as well as anthropometric and metabolic outcomes, including body weight, waist circumference, BMI and laboratory parameters.

Dr. van Maanen concluded by emphasizing that the OBA study aims to determine whether BIO101 can complement GLP‐1RA therapy by preserving muscle mass, enhancing strength and supporting broader physical and metabolic health in obese patients undergoing weight reduction.

Prof. Stephan von Haehling (University Göttingen Medical Center, Germany) concluded this session with a presentation delivered on behalf of Drs. Simon von Stengel and Wolfgang Kemmler, both exercise physiologists at the University of Nuremberg. His talk focused on a planned study investigating the role of amino acid supplementation in preserving muscle mass and mitigating rebound weight gain during and after GLP‐1RA therapy.

He started by outlining the clinical background of GLP‐1RA, which is widely used for the treatment of obesity and type 2 diabetes [37, 38]. These agents induce weight loss through mechanisms such as enhanced insulin secretion, delayed gastric emptying and appetite suppression [39]. However, he emphasized that GLP‐1‐induced weight loss is frequently accompanied by loss of lean muscle mass, often more severe than that observed with caloric restriction alone [12, 40]. Moreover, muscle mass lost during GLP‐1 therapy may not be readily regained following treatment cessation [41]. He also noted emerging concerns from preclinical data indicating that GLP‐1RA‐induced muscle loss may extend to cardiac muscle, contributing to functional decline in the setting of weight cycling [42]. These risks, coupled with relatively high discontinuation rates for GLP‐1RA therapies (about 50% in individuals with obesity and 30% in those with diabetes within 12 months) [43], underscore the need for strategies to mitigate adverse musculoskeletal effects.

To address these concerns, von Haehling introduced a proposed 12‐month, randomized, placebo‐controlled clinical trial examining two formulations of an amino acid product, which will be marketed under the name Myosamin. This product, optimized for protein synthesis and mitochondrial biogenesis, contains 95% essential amino acids, primarily branched‐chain amino acids, in a defined stoichiometric ratio. It is available in two delivery forms: a powder and a micellized liquid, both of which contain identical amino acid compositions. Myosamin has been studied over the past two decades across multiple disease states, including diabetes, heart failure, COPD and Alzheimer's disease, with consistent evidence supporting its role in improving muscle mass and function.

The proposed trial consists of three arms, each with 27 participants, all of whom have a medical indication for receiving GLP‐1RA therapy. Participants will be randomized to one of the following groups: GLP‐1RA therapy alone, GLP‐1RA therapy plus Myosamin powder, or GLP‐1RA therapy plus Myosamin micellized liquid. Standardized exercise protocols and dietary coaching will be implemented across all arms, with assessments occurring at baseline, 6 months and 12 months. Key inclusion criteria include individuals aged 30–75 years, with obesity (BMI 30–40 kg/m^2^), and no prior diagnosis of type 1 or type 2 diabetes. Exclusion criteria encompass conditions or medications that impact muscle metabolism (e.g., glucocorticoids) and agents that alter energy balance. The primary endpoints of the trial include skeletal muscle mass and total fat mass, as assessed via bioimpedance analysis. Secondary endpoints will evaluate isokinetic leg extension strength, trunk flexion and extension, sit‐to‐stand performance, 6‐min walk distance and MRI‐based assessments of muscle volume, visceral fat, myocardial mass and stroke volume. Metabolic parameters, including haemoglobin A1c, will also be tracked. To account for dietary variability, participants will complete seven‐day food records immediately before each assessment point.

Prof. von Haehling emphasized prior findings supporting the efficacy of myosamin. In a COPD trial, patients who combined exercise with Myosamin improved their 6‐min walk test performance by 110 m, compared to 27 m with exercise alone. In a follow‐up real‐world study on severely deconditioned COPD patients, those receiving Myosamin doubled their daily step count, whilst placebo recipients showed no improvement.

He concluded by suggesting that the use of essential amino acid formulations, such as Myosamin, could help preserve muscle mass, enhance physical activity and support sustained weight loss even after the cessation of GLP‐1RA therapy. Such approaches may ultimately contribute to improved functional capacity and lifestyle outcomes in individuals undergoing pharmacological weight management.

### 3.5 Discussion

The concluding discussion of this session centred on key scientific and regulatory issues related to muscle preservation, lean body mass quantification and the evolving role of combination therapies in weight loss interventions. A wide range of topics was explored, from glucagon's shifting clinical profile to the nuances of selecting meaningful endpoints for future trials.

The discussion opened with a historical reflection on the therapeutic perception of glucagon. Once regarded as harmful to diabetics due to its blood glucose‐raising effects, glucagon has undergone a dramatic re‐evaluation. As presented during the 2022 ADA meetings, glucagon is now considered beneficial in the context of obesity‐related type 2 diabetes, where it modulates core metabolic pathways. It was argued that glucagon‐centric therapies can complement GLP‐1 activity and that balanced receptor activation may improve both glycemic control and adiposity reduction without disrupting glucose homeostasis. The compound pemvidutide was designed with this balance in mind. Participants also discussed target populations for such therapies. Whilst pemvidutide is not intended for uncontrolled diabetics, it may offer particular benefits for well‐controlled diabetics and non‐diabetic individuals with obesity, especially those with fatty liver diseases like MASH.

Attention then turned to bimagrumab and its dual effects on fat loss and lean body mass. One participant noted that whilst bimagrumab has shown significant fat reduction, evidence for meaningful improvements in muscle function remains limited. Early studies largely focused on elderly populations with inclusion body myositis or sarcopenia, where functional endpoints were not achieved despite a reduction in falls. Importantly, previous studies excluded obesity participants (e.g., with a BMI over 32 kg/m^2^), leaving an evidence gap for bimagrumab's utility in broader obese populations. Observational findings from one obesity‐diabetes study suggested that bimagrumab does not suppress appetite and might even stimulate protein intake, raising hypotheses about its distinct physiological effects.

Further commentary stressed the importance of dietary protein intake. One participant cited recent work with Bruce and Bob Wolfe on essential amino acid supplementation during bariatric surgery, highlighting that essential amino acids are the most potent stimulators of muscle protein synthesis, more effective than any drug or intervention. However, in many clinical trials, reduced energy and protein intake is commonly observed, which can blunt muscle protein synthesis and thus diminish the potential impact of pharmacologic interventions. This critical factor is frequently overlooked, making nutritional strategies a vital complement.

The conversation shifted to combination therapies, with one participant raising a critical question regarding the feasibility and rationale of combining muscle anabolic agents with appetite suppressants in the treatment of obesity. In response, an example was shared of a potent anti‐myostatin agent developed by Amgen that failed in dialysis patients. The failure was attributed not to the drug's anabolic efficacy but to its inability to address the patients' profound anorexia and protein deficiency. This case underscored the necessity of integrating nutritional considerations into pharmacological interventions.

An endocrinologist specializing in obesity provided additional context, referencing the 2016 ACE Obesity Guidelines, which advocate a complication‐centric model of care. In this approach, treatment goals are aligned not with achieving arbitrary weight‐loss thresholds, but with the resolution or mitigation of obesity‐related complications such as type 2 diabetes, cardiovascular disease or sleep apnea. From this perspective, the value of preserving muscle mass lies not only in immediate functional benefits but also in long‐term health outcomes. For instance, therapies with second‐generation weight‐loss agents often result in a 2%–3% decrease in bone mass, increasing fracture risk [44]. Muscle preservation may counteract this effect and help maintain quality of life and physical activity.

Dr. Stacie Hudgens (Clinical Outcomes Solutions, Tucson, AZ, USA) emphasized the importance of carefully selecting patient‐reported outcomes (PROs) in clinical trials. She cautioned against the simultaneous use of general and disease‐specific quality‐of‐life measures, such as the SF‐36 and WQOL‐Lite, without a clear interpretive strategy. Discrepancies between results from different instruments can create ambiguity in data interpretation. Instead, she recommended pragmatic single‐item global assessments tied to functional domains relevant to the study's objectives. Such deliberate selection improves data clarity and ensures alignment with patient‐centred endpoints. She further noted that regulatory bodies, including the FDA's patient‐focused statistical support groups, may challenge the inclusion of unanchored PROs, particularly when conflicting results emerge. A regulatory representative agreed, reinforcing the principle that alpha‐protected endpoints must be contextually justified and clinically meaningful. Whilst exploratory endpoints may enrich the dataset, primary outcomes must be selected with clarity and purpose [45].

Another question was directed to Dr. Scott Harris regarding the MRI methodology used in the Momentum trial. He clarified that the lean body mass ratio was derived from a segmental MRI scan that covered the area from the thoracic vertebra T9 to the knee. According to expert advice, this region provides a representative estimate of whole‐body lean body mass. However, Harris acknowledged that the approach differs significantly from DXA‐based whole‐body assessments, creating challenges for cross‐study comparisons due to a lack of standardization and published peer‐reviewed data.

Finally, the discussion shifted towards the potential for co‐primary endpoints in trials for obesity and sarcopenia. A clinician expressed concern that conventional function‐based metrics, such as handgrip strength, may not fully capture patient‐perceived improvements in mobility and energy. They also raised the possibility that ongoing muscle loss could negatively affect long‐term metabolic outcomes, such as insulin resistance. This prompted debate over whether inflammatory biomarkers or insulin sensitivity measures could serve as valid co‐primary endpoints alongside physical function tests. A regulatory expert responded by outlining three recognized categories of endpoints: clinical (how a patient feels, functions or survives), validated surrogate endpoints (e.g., haemoglobin A1c, LDL cholesterol) and exploratory biomarkers without established links to outcomes. They acknowledged that, as with the liver and MASH fields, the path to endpoint validation involves consensus‐building across stakeholders. Guidance cannot be issued until approvable endpoints are clearly defined and widely supported.

## 4 Possible Endpoints for Regulatory Approval of Treatments Addressing Muscle Wasting in the Context of Obesity Therapy

The final session of the SCWD2024 Regulatory Workshop, led by Prof. Stefan Anker (Charité, Berlin, Germany), focused on the critical issue of endpoint selection for regulatory approval in treatments addressing muscle loss during obesity therapy. Framing the discussion, Anker acknowledged the dual aim of defining suitable endpoints and identifying pathways for future collaborative progress. He proposed that the outcomes of the current workshop be developed into a published meeting report and used as a foundation for broader consensus‐building efforts. The concept named ‘MOSAIC’ (Muscle Optimization in Obesity Initiative) was also introduced. This concept was devised by Yann Colardelle, who organizes the Cachexia Conference and Society, and aims to shape the future of clinical trials through a multidisciplinary approach and the participation of diverse stakeholders.

To stimulate discussion, Anker presented a single slide enumerating a wide array of potential clinical and surrogate endpoints. These included:

1 Mortality and Cardiovascular Mortality and Hospitalization Rates
  ○ General and cause‐specific event rates (depending on clinical context)
  ○ Hospitalizations (general or cardiovascular‐specific)
  ○ Time to first event, recurrent events or duration of events (e.g., ICU length of stay)
  ○ Faster discharge is relevant and could be a valid endpoint for some indications
  ○ All these outcomes are also safety relevant
2 Disability and Functional Limitations
  ○ Borrowing from discussions on bone mineral content in long‐term therapy, changes in bone mineral content could serve as valid measures
  ○ Quality of life and patient global assessment (global or domain‐specific):
  - EQ‐5D (for health economic assessments)
  - General and disease‐specific quality of life metrics (e.g., cancer, heart failure, CKD and COPD)

3 Functional Limitations and Reimbursement Concerns
  ○ Whilst such measures are crucial for guidelines and the medical community, cost‐effectiveness remains uncertain
4 Biomarkers (Unlikely to Gain Regulatory or Guideline Recognition)
  ○ Examples: natriuretic peptides, CRP, lipids and PQ‐2
  ○ These are more mechanistic or proof‐of‐concept markers rather than approvable endpoints
5 Sensitive Early Markers of Possible Clinical Benefit
  ○ Hand grip strength: extremely sensitive and reproducible, though approval for use remains unlikely
  ○ It could serve as a phase 2 marker for dose‐finding studies

### 4.1 Discussion

The discussion then opened among participants, who reiterated the primacy of safety, particularly in weight‐loss trials. They noted that risk–benefit considerations differ across therapeutic settings, contrasting the permissiveness of late‐stage cancer cachexia trials with the caution required for long‐term obesity interventions. One participant drew a parallel with cardiovascular outcome trials in diabetes, which were initially met with skepticism but ultimately transformed the treatment paradigm for diabetes. A similar trajectory was envisioned for sarcopenia and weight‐loss therapies if sufficient investment and consensus could be achieved.

Another participant emphasized that fat loss and muscle preservation need not be viewed in isolation. They argued that if an 8% fat loss through muscle‐sparing interventions could demonstrably improve markers such as haemoglobin A1c or steatosis, it should be considered as meaningful as the currently accepted 5% weight loss standard.

This prompted broader debate around measurement practices, with several participants noting that body fat is not routinely assessed in clinical settings. The lack of infrastructure for consistently measuring fat and muscle mass—whether through bioimpedance, plethysmography or DXA—remains a barrier. Concerns were also raised regarding the clinical standardization of functional assessments. Tools such as the SPPB (Short Physical Performance Battery) were highlighted as promising yet underutilized in daily practice.

The discussion converged on the notion that structure (muscle and fat mass), function (mobility and strength) and outcome (fractures and disability) are interconnected. Yet, because outcome‐based endpoints often require prohibitively large trials, structural and functional surrogates may be more feasible in the near term [46].

Participants further questioned whether functional endpoints such as stair‐climbing ability should be necessary in diseases defined by excess adiposity. If obesity is clinically understood as a disease of excess fat, demonstrating fat reduction may be sufficient for approval. Reference was made to ABCD (Adiposity‐Based Chronic Disease) guidelines, which prioritize fat mass over muscle mass. In this view, fat mass is easier to measure than muscle mass, and future guidelines may increasingly reflect this reality.

The session closed with broad agreement that integrating body composition measures into routine clinical trials could significantly shift both clinical practice and regulatory evaluation [47]. Incorporating routine assessments of body composition has the potential to fundamentally alter the therapeutic paradigm. At present, a critical gap remains between mechanistic insight and actionable clinical outcomes—a gap that future studies and collaborative frameworks such as MOSAIC must strive to bridge.

## 5 Conclusions

The latter half of the December 2024 SCWD Regulatory Workshop focused on incretin‐based anti‐obesity therapies and their implications for skeletal muscle health, functional capacity and the design of future regulatory pathways. GLP‐1RA such as semaglutide and tirzepatide have demonstrated remarkable efficacy in weight reduction; however, concern was raised about the disproportionate loss of lean body mass observed in clinical trials. Speakers highlighted the critical distinction between total lean body mass and ‘true’ skeletal muscle, pointing out that DEXA may overestimate lean body mass loss by including non‐contractile compartments. Advanced imaging methods such as MRI and CT were recognized as necessary to refine the measurement of contractile muscle volume, adipose depots and intramuscular fat infiltration, although the lack of methodological standardization remains a barrier to broader implementation.

A central theme was the long‐term trajectory of muscle mass and function after anti‐obesity treatment discontinuation. Whilst weight regain is common following the withdrawal of GLP‐1 therapies, available evidence does not clearly support preferential fat regain over muscle. Nevertheless, older adults face a distinct challenge: their limited ability to restore skeletal muscle after weight loss increases the risk of sarcopenia and frailty. Participants agreed that safety and efficacy in this vulnerable population deserve urgent investigation. This need is underscored by evidence of their cardiovascular and renal benefits, which has led to an expansion of their use into beyond middle‐aged cohorts.

Therapeutic strategies aimed at preserving muscle during weight reduction were explored in depth. Several agents currently in clinical or preclinical testing—including bimagrumab, a monoclonal antibody targeting activin type 2 receptors; pemvidutide, a dual GLP‐1 and glucagon receptor agonist; enobosarm, a selective androgen receptor modulator; BIO101 (20‐hydroxyecdysone); and amino acid supplementation (Myosamin)—were presented as promising adjuncts to standard GLP‐1 therapy. These approaches share the common goal of mitigating lean body mass decline whilst amplifying metabolic benefits such as fat loss and glycaemic control. However, experts also noted that improvements in lean body mass do not automatically translate into better strength or mobility, underscoring the need to target both structural and functional domains simultaneously.

From a regulatory perspective, the discussions converged on the importance of defining clinically meaningful outcomes. Structural endpoints (muscle and fat mass), functional endpoints (mobility and strength), and clinical outcomes (fractures and disability) are inherently interrelated, whilst outcome‐based endpoints often necessitate prohibitively large and lengthy trials. As such, structural and functional measures may serve as pragmatic surrogates in the short term, provided they are standardized and validated. Patient‐reported outcomes were also highlighted as essential to capture real‐world benefits that matter to patients, including independence and confidence in daily living.

Finally, the workshop emphasized, with broad consensus, that the integration of body composition assessments into routine clinical trials, combined with standardization of functional testing protocols, could substantially shift both clinical practice and regulatory assessment. These steps are seen as critical for bridging the current gap between mechanistic insight and approvable clinical outcomes. The emerging ‘MOSAIC’ (Muscle Optimization in Obesity Initiative) framework was noted as a potential vehicle for consensus‐building across academia, industry and regulators.

## Funding

The authors received no specific funding for this work.

## Conflicts of Interest

The authors declare no conflicts of interest.
